# Correlated physical and mental health composite scores for the RAND-36 and RAND-12 health surveys: can we keep them simple?

**DOI:** 10.1186/s12955-022-01992-0

**Published:** 2022-06-03

**Authors:** John Roger Andersen, Kyrre Breivik, Inger Elise Engelund, Marjolein M. Iversen, Jorunn Kirkeleit, Tone Merete Norekvål, Kjersti Oterhals, Anette Storesund

**Affiliations:** 1 Department of Research and Development, Centre on Patient-Reported Outcomes, Bergen Hospital Trust, Bergen, Norway; 2 Department of Health and Caring Sciences, Faculty of Health and Social Sciences, Western Norway University of Applied Sciences, Bergen/Førde, Norway; 3 Regional Centre for Child and Youth Mental Health and Child Welfare, NORCE, Norwegian Research Centre, Bergen, Norway; 4 Department of Global Public Health and Primary Care, University of Bergen, Bergen, Norway; 5 Department of Clinical Science, University of Bergen, Bergen, Norway; 6 Department of Heart Disease, Haukeland University Hospital, Bergen, Norway; 7 Førde Hospital Trust, Førde, Norway

**Keywords:** RAND-36, RAND-12, SF-36, SF-12, PCS, MCS, Oblique, Unweighted, Psychometric properties, Validity

## Abstract

**Background:**

The RAND-36 and RAND-12 (equivalent to versions 1 of the SF-36 Health Survey and SF-12 Health Survey, respectively) are widely used measures of health-related quality of life. However, there are diverging views regarding how to create the physical health and mental health composite scores of these questionnaires. We present a simple approach using an unweighted linear combination of subscale scores for constructing composite scores for physical and mental health that assumes these scores should be free to correlate. The aim of this study was to investigate the criterion validity and convergent validity of these scores.

**Methods:**

We investigated oblique and unweighted RAND-36/12 composite scores from a random sample of the general Norwegian population (*N* = 2107). Criterion validity was tested by examining the correlation between unweighted composite scores and weighted scores derived from oblique principal component analysis. Convergent validity was examined by analysing the associations between the different composite scores, age, gender, body mass index, physical activity, rheumatic disease, and depression.

**Results:**

The correlations between the composite scores derived by the two methods were substantial (r = 0.97 to 0.99) for both the RAND-36 and RAND-12. The effect sizes of the associations between the oblique versus the unweighted composite scores and other variables had comparable magnitudes.

**Conclusion:**

The unweighted RAND-36 and RAND-12 composite scores demonstrated satisfactory criterion validity and convergent validity. This suggests that if the physical and mental composite scores are free to be correlated, the calculation of these composite scores can be kept simple.

## Background

The RAND-36, and its brief version, the RAND-12 (equivalent to version 1 of the SF-36 Health Survey and SF-12 Health Survey, respectively), are freely available and widely used measures of generic health-related quality of life (HRQoL) [1–4]. HRQoL refers to “how health impacts on an individual’s ability to function and his or her perceived well-being in physical, mental and social domains of life” [3]. The RAND-36/12 provides data on eight subscale scores and two composite scores of physical and mental health. The use of composite scores has become quite popular as they can simplify the interpretation of the findings [2]. However, despite the widespread use of the RAND-36/12 composite scores, the choice of method for constructing them has been a controversial issue for decades [5–10].

Originally, Ware et al. [2, 11] provided algorithms for constructing composite scores based on orthogonal principal component analysis (PCA) to create a physical composite summary (PCS) and a mental composite summary (MCS) for version 1 of the SF-36/12 Health Surveys. They aimed to create pure PCS and MCS scores with little overlapping variance. To achieve this, all the scales/items must be included in the two composite scores but have different weights. However, this orthogonal approach has been criticized for producing inconsistencies between the composite scores and the observed data [5–7, 9].

Thus, a range of alternative scoring algorithms has been developed that do not restrict the correlations between the PCS and MCS [12]. One of the best documented alternatives to the orthogonal PCS and MCS was published by Farivar et al. in 2007, using oblique PCA to create the RAND-36/12 composite scores, which allowed correlations between them [7]. Overall, approaches such as this seem to be less prone to produce inconsistencies with the observed data [5–7].

On the other hand, a correlated PCS and MCS might not be without limitations. For example, a PCS and MCS from oblique PCA tend to be very strongly correlated, inducing multicollinearity [8]. Another issue is that the weights from oblique PCA fluctuate according to sample characteristics, making standardization across samples problematic [12, 13]. Furthermore, several authors have advocated the use of weights from confirmatory factor analysis (CFA) to create a PCS and MCS that are permitted to correlate [14, 15]. However, using CFA to construct composite scores can be problematic from a theoretical point of view, as a composite score, by nature, is a multidimensional construct [13, 16].

Hence, there are many alternatives to an orthogonal PCS and MCS for the RAND-36/12, making it unclear for researchers to decide which one to use [6, 12]. It has been argued that we often tend to make HRQoL scores unnecessarily complicated, for example, by using weighted scores [17]. Thus, simple unweighted composite scores have been proposed for the RAND-36/12. Such simple composite scores show promising criterion validity [5, 18]. This is not surprising given the strong correlations among the indicators of the RAND-36/12 composite scores. Weighting is probably of little value under such conditions [19], a logic that also applied when the original developers decided not to use weights for the SF-36 subscores [20]. However, data on the convergent validity for the unweighted RAND-36/12 is lacking in studies that have used unweighted composite scores for them [5, 18]. This is a limitation, as convergent validity is a crucial part of evaluating psychometric properties [21].

We present a simple approach to construct unweighted composite scores for the RAND-36/12, which implicitly assumes that these scores should be allowed to correlate. The composite scores were created by a linear combination of (1) The four subscales that have shown to be primarily indicative of physical health (physical functioning, physical role functioning, bodily pain and, general health), and (2) The four subscales that have shown to be primarily indicative of mental health (vitality, social functioning, emotional role functioning, and mental health). The aim of this study was to investigate the criterion and convergent validity of these scores by comparing them to established oblique composite scores. We hypostatized that unweighted and oblique composite scores would be highly correlated with each other and demonstrate equal convergent validity.

## Methods

### Design and study participants

We reused data from a representative survey of the general population of Norwegian adults aged 18–79 years. The methods have been described in detail previously [22]. In brief, the sample consisted of 2107 persons (36% response rate) who completed the Norwegian version of the RAND-36 (equivalent to the SF-36 version 1) as a postal questionnaire in 2015. All the items in the RAND-12 were taken directly from the RAND-36.

### Demographic and other variables

We included self-reported data on age (10-year intervals), gender (women, men), marital/cohabitation status (no, yes), education (elementary school, high school, university < 4 years, and university ≥ 4 years), strenuous physical activity habits (never, less than 1 h per week, 1–2 h per week, and ≥ 3 h per week), self-reported height and weight (body mass index), and self-reported history of being diagnosed with a rheumatic disease or depression (no, yes) [22].

### RAND-36 measures and scoring

Oblique RAND-36 PCS and MCS composite scores were created using the method, including the scoring coefficients described by Farivar et al. [7]. First, all the items were standardized into 0–100 scores. Second, eight subscales were created based on the mean scores of items belonging to the same scale: physical functioning (10 items), physical role functioning (4 items), bodily pain (2 items), general health (5 items), vitality (4 items), social functioning (2 items), emotional role functioning (3 items), and mental health (5 items). The subscale scores ranged from 0 to 100, with higher scores indicating better HRQoL. Third, eight z-scores were made using the mean and standard deviations of SF-36 subscales from a 1998 US norm population, described in the manual of Ware et al. [2]. Forth, the z-scores were weighted using published scoring coefficients from oblique factor analysis. The only difference from the method of Ware et al. [2] and the present one is that we weighted the composite scores based on published scoring coefficients derived from the oblique PCA analysis in the study of Farivar et al. [7]. Finally, T-scores were made with a mean of 50 (SD = 10) representing the average scores in the US norm population [2].

The unweighted RAND-36 PCS and MCS composite scores were based on the original subscales, ranging from 0 to 100. Previous studies have shown that four subscale scores predominantly reflect physical health, while four others predominantly reflect mental health [6]. Thus, the unweighted RAND-36 PCS was created by adding the subscale scores for physical functioning, physical role functioning, bodily pain, and general health and dividing the sum by 4. The unweighted RAND-36 MCS was created by adding the subscale scores for vitality, social functioning, emotional role functioning, and mental health and dividing the sum by 4. This is quite similar to the RAND-HSI scoring but without the weights [6]. The unweighted RAND-36 PCS and MCS ranged from 0 to 100, with higher scores indicating better HRQoL.

### RAND-12 measures and scoring

The oblique RAND-12 PCS and MCS composite scores were also created by the method of Farivar et al. [7]. The scoring was based on regressing the oblique RAND-36 PCS and MCS T-scores in separate models for the RAND-12 items. From these results, weighted dummy variables were used to create RAND-12 PCS and MCS T-scores, with higher scores indicating better HRQoL.

The unweighted RAND-12 PCS and MCS composite scores were created by standardizing the 12-items to 0–100 scores, in the same way as done for the RAND-36. Eight subscales were created, based on mean scores of items belonging to the same scale: physical functioning (2 items), physical role functioning (2 items), bodily pain (1 item), general health (1 item), vitality (1 item), social functioning (1 item), emotional role functioning (2 items), and mental health (2 items). Subscale scores ranged from 0 to 100, with higher scores indicating better HRQoL. The unweighted RAND-12 PCS score was created by adding the subscale scores for physical functioning, physical role functioning, bodily pain, and general health and dividing the sum by 4. The unweighted RAND-12 MCS score was created by adding the subscale scores for vitality, social functioning, emotional role functioning, and mental health and dividing the sum by 4. The unweighted RAND-12 PCS and MCS scores ranged from 0 to 100, with higher scores indicating better HRQoL.

### Statistics

Characteristics of the study population are presented as means and standard deviations or raw numbers and percentages. Descriptive statistics of the RAND-36/12 composite scores were based on z-scores (means = 0 and standard deviations = 1) because the different versions of the composite scores are not on the same metric. We present features of score distributions by medians, modes, floor, and ceiling effects (percentages), item-total correlations corrected for overlap, along with values for skewness and kurtosis. Floor or ceiling effects might be a problem if ≥ 15% of respondents obtain the worst or best possible score [23]. The corrected item-total correlations were calculated for the unweighted RAND-36/12 composite scores: PCS (physical functioning, physical role functioning, bodily pain, and general health) and MCS (vitality, social functioning, emotional role functioning, and mental health), with values ≥ 0.4 indicating scores that consist of highly correlated variables [20]. Values of skewness ≥ 2 and kurtosis ≥ 7 suggest distributions of scores that begin to depart substantially from normality [24]. Associations between subscale scores and the PCS and MCS composite scores were examined using Pearson correlations or Spearman rank correlations. Criterion validity was examined by Pearson correlations between unweighted composite scores and scores derived from the oblique factor scoring coefficients [7]. Based on previous research and theory, the correlations between the unweighted and oblique RAND-36/12 composite scores measuring the same construct should be ≥ 0.95 [5, 18, 21]. Convergent validity was examined using Spearman rank coefficients between the composite scores and variables known to be related to HRQoL: age (years, continuous); sex (women = 0, men = 1); body mass index (units, continuous); physical activity (strenuous physical activity: never = 0, less than 1 h per week = 1, 1–2 h per week = 2, ≥ 3 h per week = 3), rheumatic disease (no = 0, yes = 1), and depression (no = 0, yes = 1)[2, 11, 25–28]. A correlation ≥ 0.2 regarding convergent validity suggest an effect size that might be of practical importance [29]. SPSS version 27 was used to perform the statistical analyses (IBM Corporation).

## Results

The characteristics of the study participants are presented in Table 1. Table 2 shows that the means and standard deviations of unweighted RAND-36 versus RAND-12 scores (0–100) were not directly comparable, with the RAND-12 composite scores being somewhat lower. Descriptive statistics of the RAND-36/12 composite z-scores and features of score distributions showed approximately similar results for unweighted and oblique scores (Table 3). An exception was that the unweighted RAND-12 compositive scores had slightly higher ceiling values, although none of them were above ≥ 15%. The corrected item-total correlations for the unweighted RAND-36/12 composite scores ranged from 0.52 to 0.74, indicating scores that consist of highly correlated variables. The correlations between the RAND-36/12 composite scores and the respective subscale scores showed a pattern with oblique PCS scores having stronger correlations with the subscales representing mental health and vice versa (Tables 4–5). The correlations between the composite scores derived from the two methods were very strong (r = 0.97 to 0.99) for both the RAND-36 and RAND-12 (Table 6). The correlations between the PCS and MCS derived by the two methods were weaker for the unweighted method than for the oblique method for both the RAND-36 (r = 0.61 vs. r = 0.78) and RAND-12 (r = 0.58 vs. r = 0.79). The effect sizes of the associations between the oblique versus unweighted composite scores and other variables had comparable magnitudes, indicating similar convergent validity (Table 7).

**Table 1:** Characteristics of the study population (*n* = 2107)

Variables	Values
Age in years, n (%)	
18–29	105 (5.0)
30–39	203 (9.6)
40–49	400 (19.0)
50–59	484 (23.0)
60–69	519 (24.6)
70–79	396 (18.8)
Gender, n (%)	
Women	1143 (54.8)
Men	943 (45.2)
Married or cohabiting, n (%)	
Yes	1603 (76.1)
No	504 (23.9)
Education, n (%)	
Elementary school	79 (18.0)
High School	777 (37.0)
University < 4 years	457 (21.8)
University ≥ 4 years	486 (23.2)
Strenuous physical activity, n (%)	
Never	292 (17.4)
Less than 1 h per week	375 (22.4)
1–2 h per week	596 (35.6)
≥ 3 h per week	410 (24.5)
Body mass index, mean (SD)	26.1 (4.5)
History of rheumatic disease, n (%)	
No	1766 (92.3)
Yes	148 (7.7)
History of depression, n (%)	
No	1656 (86.1)
Yes	268 (13.9)

Variables with fewer than 2017 observations are due to missing data

**Table 2:** Unweighted RAND 36/12 composite scores and subscores (0–100) presented with means and standard deviations

Scores	RAND-36	RAND-12
*Physical Composite Summary*	77.4 (21.5)	75.1 (23.4)
Physical functioning	87.3 (18.3)	86.9 (23.9)
Physical role functioning	76.8 (37.2)	74.4 (39.9)
Bodily pain	73.7 (25.1)	81.6 (25.1)
General health	71.8 (21.3)	57.3 (23.9)
*Mental Composite Summary*	79.4 (16.6)	75.1 (18.9)
Vitality	59.6 (19.9)	48.5 (26.1)
Social functioning	87.9 (20.2)	86.7 (22.2)
Emotional role functioning	88.9 (26.9)	87.7 (29.2)
Mental health	81.0 (14.3)	77.3 (18.6)

**Table 3:** Descriptive statistics of the RAND-36/12 compositive z-scores (means = 0 and standard deviations = 1) and features of score distributions

RAND-36 subscales	RAND-36 PCS unweighted	RAND-36 PCS oblique	RAND-36 MCS unweighted	RAND-36 MCS oblique	RAND-12 PCS unweighted	RAND-12 PCS oblique	RAND-12 MCS unweighted	RAND-12 MCS oblique
Median	0.40	0.37	0.33	0.25	0.55	0.37	0.28	0.23
Mode	1.00	1.20	0.62	1.53	0.82	0.82	0.67	0.78
Skewness	− 1.10	− 1.10	− 1.63	− 1.21	− 1.09	− 1.11	− 1.24	− 1.08
Kurtosis	0.20	0.38	2.60	1.59	0.15	0.40	1.28	1.11
% Floor	0	0	0	0	0.1	0.1	0.1	0.1
% Ceiling	2.7	0.7	1.5	0.7	8.3	1.3	3.9	1.3
Corrected item-total correlation	PF = 0.70 RP = 0.74 BP = 0.69 GH = 0.68	NA	VT = 0.63 SF = 0.71 RE = 0.58 MH = 0.72	NA	PF = 0.66 RP = 0.72 BP = 0.71 GH = 0.61	NA	VT = 0.58 SF = 0.62 RE = 0.52 MH = 0.68	NA

*PCS* Physical Composite Summary; *MCS* Mental Composite Summary.; *PF* physical functioning, *RP* physical role functioning, *BP* bodily pain, *GH* general health, *VT* vitality, *SF* social functioning, *RE* role functioning, *MH* mental health, *NA* not applicable

**Table 4:** Pearson correlations between RAND-36 subscales and unweighted and oblique composite scores

RAND-36 subscales	PCS unweighted	PCS oblique	MCS unweighted	MCS oblique
Physical functioning	0.80	0.77	0.44	0.47
Physical role functioning	0.90	0.88	0.52	0.56
Bodily pain	0.83	0.82	0.49	0.57
General health	0.80	0.82	0.61	0.71
Vitality	0.61	0.72	0.79	0.88
Social functioning	0.60	0.69	0.85	0.80
Emotional role functioning	0.42	0.48	0.82	0.70
Mental health	0.38	0.46	0.82	0.86

*PCS* Physical Composite Summary; *MCS* Mental Composite Summary

**Table 5:** Spearman rank correlations between the RAND-12 subscales and unweighted and oblique RAND-12 composite scores

RAND-12 subscales	PCS unweighted	PCS oblique	MCS unweighted	MCS oblique
Physical functioning	0.72	0.66	0.40	0.43
Physical role functioning	0.81	0.78	0.48	0.53
Bodily pain	0.79	0.79	0.49	0.56
General health	0.82	0.75	0.52	0.59
Vitality	0.55	0.73	0.86	0.86
Social functioning	0.53	0.63	0.73	0.68
Emotional role functioning	0.38	0.44	0.62	0.56
Mental health	0.32	0.50	0.79	0.83

*PCS* Physical Composite Summary; *MCS* Mental Composite Summary

**Table 6:** Pearson correlations between the RAND-36/12 composite scores

	RAND-36 PCS unweighted	RAND-36 PCS oblique	RAND-12 PCS unweighted	RAND-12 PCS oblique	RAND-36 MCS unweighted	RAND-36 MCS oblique	RAND-12 MCS unweighted	RAND-12 MCS oblique
RAND-36 PCS unweighted	1							
RAND-36 PCS oblique	**0.99**	1						
RAND-12 PCS unweighted	0.97	0.96	1					
RAND-12 PCS oblique	0.96	0.98	**0.98**	1				
RAND-36 MCS unweighted	0.61	0.71	0.60	0.73	1			
RAND-36 MCS oblique	0.68	0.78	0.66	0.79	**0.97**	1		
RAND-12 MCS unweighted	0.59	0.69	0.58	0.72	0.97	0.96	1	
RAND-12 MCS oblique	0.66	0.75	0.66	0.79	0.94	0.97	**0.97**	1

Correlations between scores reflecting the same construct are marked in bold

*PCS* Physical Composite Summary; *MCS* Mental Composite Summary

**Table 7:** Spearman rank correlations between RAND-36/12 composite scores and related variables

	Age	Sex	Body mass index	Physical Activity	Rheumatic disease	Depression
RAND-36 PCS unweighted	− 0.21	0.06	− 0.21	0.27	− 0.26	− 0.21
RAND-36 PCS oblique	− 0.13	0.08	− 0.20	0.27	− 0.26	− 0.24
RAND-12 PCS unweighted	− 0.25	0.06	− 0.23	0.30	− 0.24	− 0.20
RAND-12 PCS oblique	− 0.12	0.10	− 0.19	0.29	− 0.23	− 0.25
RAND-36 MCS unweighted	0.11	0.10	− 0.07	0.20	− 0.14	− 0.33
RAND-36 MCS oblique	0.10	0.10	− 0.09	0.22	− 0.17	− 0.33
RAND-12 MCS unweighted	0.10	0.11	− 0.07	0.21	− 0.15	− 0.32
RAND-12 MCS oblique	0.09	0.11	− 0.09	0.23	− 0.17	− 0.32

Age (years, continuous); sex (women = 0, men = 1), body mass index (units, continuous), physical activity (strenuous physical activity: never = 0, less than 1 h per week = 1, 1–2 h per week = 2, ≥ 3 h per week = 3), rheumatic disease (no = 0**,** yes = 1), depression (no = 0, yes = 1)

## Discussion

We found strong correlations between the composite scores derived by the two methods for both the RAND-36 and RAND-12 and that the effect sizes of the associations between the oblique versus the unweighted composite scores and other variables had comparable magnitudes, also indicating similar convergent validity. The features of score distributions of the corresponding composite scores showed approximately similar results, except for unweighted RAND-12 composite scores having slightly higher ceiling effects.

To the best of our knowledge, this is the first study to report both the criterion validity and convergent validity of unweighted RAND-36/12 composite scores. However, two prior studies have reported the criterion validity of the RAND-36 or RAND-12 composite scores using two other methods for constructing unweighted scores. Grassi et al. [30] used data from the European Community Respiratory Health Survey and compared SF-36 composite scores derived from oblique PCA with those from an unweighted scoring system. The unweighted PCS was calculated as the sum of 18 items, while the MCS included 19 items. The correlation between the oblique and unweighted PCS was 0.97, and 0.96 between oblique and unweighted MCS. The correlation between the unweighted PCS and MCS was 0.61.

Hagell et al. [5] applied data from people with Parkinson’s disease and stroke to compare SF-12 composite scores derived from the RAND-12 HSI algorithm that produced similar results to scores based on oblique PCA. The unweighted PCS was calculated as the raw sum of six items, while the MCS was from six other distinct items. The correlation between the weighted and unweighted scores were 0.99 for both PCS and MCS. The correlation between the unweighted PCS and MCS was 0.68.

The scoring methods in these two studies differed slightly from ours by using the sum of items to create raw scores, while we used unweighted linear combinations of subscale scores based on items that were standardized, ranging from 0 to 100. We think that a two-step method that initially scores the subscales and then uses them to create composite scores is more intuitive, considering that the subscales have a different number of items. However, the practical difference between our approach and the two other unweighted approaches for scoring composite scores seems to be minor. These findings are not surprising, given the strong correlations between the items that contribute to the RAND-36/12 composite scores.

We found that the correlations between the unweighted RAND-36/12 PCS and MCS were weaker than those created from oblique PCA. A reason for this is that oblique PCA produces weights for creating PCS and MCS that increase the correlation between these scores [7]. In the unweighted approach, no restraints are imposed, and the PCS and MCS are completely free to correlate. This could be a strength favouring unweighted RAND-36/12 composite scores, as correlations approaching 0.80 may induce multicollinearity if the PCS and MCS are used as independent variables in the same model [31].

Regarding convergent validity, the associations between the oblique versus the unweighted RAND-36/12 composite scores and other variables had comparable magnitudes. An exception was that age was more strongly correlated with the unweighted PCS scores than the oblique ones. This could reflect that the oblique PCS scores were based on all subscales being either negatively, neutral, or positively correlated with age. There also seems to be a subtle tendency for the oblique PCS and MCS to have more similar effect sizes than the unweighted PCS and MCS. This probably reflects the stronger correlations between the oblique PCS and MCS.

The strengths of this study include a sufficiently large sample from a general population, and that convergent validity was examined. A limitation of the study is that weight, height, physical activity, rheumatic disease, and depression were assessed by self-reports. However, the included measures have been shown to have acceptable validity [32–34]. Second, our cross-sectional design did not allow us to study longitudinal changes in unweighted versus weighted composite scores. Thus, differences in the responsiveness of change should be explored in future studies.

The main implication of this study is that we can keep the calculation of the RAND-36/12 composite scores simple. This has several advantages, such as the standardization of scoring across studies and populations. In this paper, we calculated composite scores ranging from 0 to 100, but the data can easily be converted to T-scores. An advantage of the previous scoring approaches is good comparability between PCS-12 and PCS-36 and between MCS-12 and MCS-36 [2, 7]. Such comparability is not seen with our current proposed scoring method. However, similar comparability could be derived by regressing the unweighted RAND-36 PCS and MCS T-scores in separate models for the two unweighted RAND-12 composite scores. This could be explored in a future study using a cross-validated design. It should be emphasized that our findings do not imply that weighted composite scores of HRQoL are never useful or that prior studies using different oblique composite scores for the RAND-36/12 have led to erroneous results. However, we propose that weighting is likely to be redundant if analyses of composite scores show corrected item-total correlations values ≥ 0.4. This knowledge should also be useful to consider when developing composite scores for new HRQoL instruments.

## Conclusions

In conclusion, the unweighted RAND-36/12 composite scores demonstrated satisfactory validity. Consequently, the calculation of these composite scores can be kept simple when we want them to be free to correlate. Future studies should examine the external validity of our findings and the sensitivity of the change in unweighted versus weighted composite scores in different populations.

## Data Availability

The data is owned by a third party: Marianne Jensen Hjermstad, Kjersti S. Grotmol and Håvard Loge (Regional Advisory Unit for Palliative Care, Dept. of Oncology, Oslo University Hospital, Norway). E-mail: mariajhj@medisin.uio.no. Data are however available from the corresponding author upon reasonable request and with permission of the third party.

## Abbreviations

CFA: Confirmatory factor analysis
HRQoL: Health-related quality of life
MCS: Mental composite summary
PCA: Principal component analysis
PCS: Physical composite summary
RAND: Research and development
